# Frequent Prescription of Antibiotics and High Burden of Antibiotic Resistance among Deceased Patients in General Medical Wards of Acute Care Hospitals in Korea

**DOI:** 10.1371/journal.pone.0146852

**Published:** 2016-01-13

**Authors:** Yee Gyung Kwak, Chisook Moon, Eu Suk Kim, Baek-Nam Kim

**Affiliations:** 1 Department of Internal Medicine, Inje University Ilsan Paik Hospital, Goyang, Republic of Korea; 2 Department of Internal Medicine, Inje University Busan-Paik Hospital, Busan, Republic of Korea; 3 Department of Internal Medicine, Seoul National University Bundang Hospital, Seongnam, Republic of Korea; 4 Department of Internal Medicine, Inje University Sanggye-Paik Hospital, Seoul, Republic of Korea; University of Iowa Carver College of Medicine, UNITED STATES

## Abstract

**Background:**

Antibiotics are often administered to terminally ill patients until death, and antibiotic use contributes to the emergence of multidrug-resistant organisms (MDROs). We investigated antibiotic use and the isolation of MDROs among patients who died in general medical wards.

**Methods:**

All adult patients who died in the general internal medicine wards at four acute care hospitals between January and June 2013 were enrolled. For comparison with these deceased patients, the same number of surviving, discharged patients was selected from the same divisions of internal medicine subspecialties during the same period.

**Results:**

During the study period, 303 deceased patients were enrolled; among them, 265 (87.5%) had do-not-resuscitate (DNR) orders in their medical records. Antibiotic use was more common in patients who died than in those who survived (87.5% vs. 65.7%, P<0.001). Among deceased patients with DNR orders, antibiotic use was continued in 59.6% of patients after obtaining their DNR orders. Deceased patients received more antibiotic therapy courses (two [interquartile range (IQR) 1–3] vs. one [IQR 0–2], P<0.001). Antibiotics were used for longer durations in deceased patients than in surviving patients (13 [IQR 5–23] vs. seven days [IQR 0–18], P<0.001). MDROs were also more common in deceased patients than in surviving patients (25.7% vs. 10.6%, P<0.001).

**Conclusions:**

Patients who died in the general medical wards of acute care hospitals were exposed to more antibiotics than patients who survived. In particular, antibiotic prescription was common even after obtaining DNR orders in patients who died. The isolation of MDROs during the hospital stay was more common in these patients who died. Strategies for judicious antibiotic use and appropriate infection control should be applied to these patient populations.

## Introduction

Antibiotic use contributes to increased antibiotic resistance due to the selection and expression of antibiotic resistance genes in bacterial populations [1]. Antibiotic abuse can result in antibiotic resistance in individual patients. Antibiotic resistance has been associated with increased attributable length of hospital stay, mortality, and health care costs [2].

Physicians and family members often consider antibiotics to be a minimum treatment requirement for terminally ill patients, and they are often administered until death in those patients [3–5]. As in other Asian countries where palliative and hospice care services are not widely utilized for patients with terminal illnesses [6,7], most terminally ill patients in Korea are admitted to acute care hospitals near the end of their lives in order to receive life-sustaining treatments [7].

A recent investigation of intensive care units (ICUs) reported that dying patients without withdraw orders received more antibiotics and developed more multidrug-resistant organisms (MDROs) [8]. Patients who acquire MDROs before death may serve as MDRO reservoirs, transmitting the organisms to surviving patients in the hospital setting [8,9].

While there have been many studies on antibiotic use and resistance in critically ill patients in ICUs and in patients who die in palliative and hospice care settings [3–5,10–12], scant attention has been paid to antibiotic use in patients who die in the general hospital wards. We hypothesized that a certain proportion of patients who died in the general medical wards of acute care hospitals might be exposed to antibiotics before death, and that the isolation of MDROs might be common in these patients during their hospital stay. The aim of this study was to examine the antibiotic use and isolation of MDROs among patients who died in the general medical wards of acute care hospitals and to compare these characteristics with those of surviving, discharged patients.

## Materials and Methods

### Design

This retrospective study was conducted at four university-affiliated acute care hospitals in Korea. The study protocol was approved by the Institutional Review Board (IRB) of the Inje University Sanggye-Paik Hospital (SPIRB 13–037) as the central IRB for this multicenter study, which waived the need for written or oral informed consent from the participants.

### Subjects

All patients ≥18 years of age who died in the internal medicine wards between January and June 2013 were enrolled. For comparison with these deceased patients, we also enrolled the same number of surviving patients discharged from the same divisions of the internal medicine subspecialties during the same study period with differences in length of hospital stay ≤5 days. Patients who were hospitalized ≤2 days or >60 days, were transferred to other hospitals, or were discharged against medical advice were excluded.

### Data collection

Medical records were reviewed retrospectively, and data were collected using standardized case report forms. The data obtained included demographic characteristics, length of hospital stay, underlying diseases or conditions, and either clinical or microbiological bacterial infection during the admission and at the time of death or discharge. Data on antibiotic exposure for more than 24 hours and isolation of MDROs during the admission were also collected. An antibiotic therapy course was defined as the use of different antibiotics for at least one day during admission. Days of antibiotic use were defined as the total number of days that a single antibiotic (parenteral or oral) was administered, regardless of dosage [13]. MDROs in this study included methicillin-resistant *Staphylococcus aureus*, vancomycin-resistant enterococci, third-generation cephalosporin-resistant Enterobacteriaceae, and carbapenem-resistant non-fermenters, including *Acinetobacter baumannii* and *Pseudomonas aeruginosa* [14]. In addition, a do-not-resuscitate (DNR) order was defined as present if the patient had a preprinted DNR form or recorded verbal communication in their medical record [15].

### Statistical analysis

Bivariable analysis was performed separately for each variable. P values were calculated using chi-square or Fisher’s exact tests for categorical variables. Continuous variables were compared using Student’s *t* (for normally distributed variables) or Mann—Whitney *U* tests (for non-normally distributed variables). P values <0.05 were considered statistically significant. All variables that were statistically significant in bivariable analyses were included in logistic regression analysis. Results from the multivariable analysis are presented as adjusted odds ratios (AORs) with 95% confidence intervals (CIs).

## Results

During the study period, a total of 15,777 patients were discharged from the general internal medicine wards of four hospitals, while 377 died in the hospital. Of these deceased patients, 74 were excluded, and the remaining 303 comprised the study population (Table 1). The same number of surviving patients was selected from among the 15,400 surviving patients who were discharged. Overall, the most common underlying disease was solid tumor (75.6% vs. 58.7%, P<0.001), followed by cardiovascular disease (42.9% vs. 34.7%, P = 0.037) and diabetes mellitus (27.4% vs. 24.8%).

**Table 1:** Comparison of baseline characteristics of deceased and surviving patients in general medical wards.

Variable	Died patients (N = 303)	Survived patients (N = 303)	P value
**Male (%)**	187 (61.7)	178 (58.7)	0.455
**Age, years median (IQR)**	68 (55–76)	65 (56–74)	0.092
**Underlying disease (%)**			
solid tumor	229 (75.6)	178 (58.7)	<0.001
cardiovascular disease	130 (42.9)	105 (34.7)	0.037
diabetes mellitus	83 (27.4)	75 (24.8)	0.459
neurologic disease	50 (16.5)	32 (10.6)	0.033
chronic liver disease	49 (16.2)	47 (15.5)	0.824
chronic obstructive pulmonary disease	35 (11.6)	28 (9.2)	0.352
hematologic disease	25 (8.3)	35 (11.6)	0.174
biliary/pancreatic disease	19 (6.3)	19 (6.3)	1.000
end-stage renal disease	15 (5.0)	13 (4.3)	0.699
connective tissue disease	8 (2.6)	4 (1.3)	0.383
organ/hematopoietic transplantation	1 (0.3)	1 (0.3)	1.000
**Predisposing condition (%)**			
indwelling urethral catheter	136 (44.9)	37 (12.2)	<0.001
central venous catheter	74 (24.4)	49 (16.2)	<0.012
anticancer chemotherapy	69 (22.8)	108 (35.6)	<0.001
nasogastric or gastrostomy tube	61 (20.1)	11 (3.6)	<0.001
immunosuppressive therapy	22 (7.3)	49 (16.2)	0.001
neutropenia	25 (8.3)	32 (10.6)	0.330
biliary drainage tube	14 (4.6)	11 (3.6)	0.540
surgery	10 (3.3)	23 (7.6)	0.030
renal replacement therapy	5 (1.7)	7 (2.3)	0.560
tracheostomy tube	3 (1.0)	2 (0.7)	0.653

IQR, interquartile range.

Of the 303 deceased patients, 265 (87.5%) had DNR orders in their medical records (Table 2). On average, the first record of DNR order occurred on the 12th day (median, interquartile range [IQR] 4–21) of a 14-day hospital stay (median, IQR 8–26). The median time from DNR order to death was two (IQR 1–6) days, and 53 patients (19.6%) had DNR orders on the day of their death. Among deceased patients with DNR orders, 59.6% continued to receive antibiotics after providing a DNR order. Antibiotics were administered for a median of two (IQR 0–5) days after the day when DNR consent was provided in those patients. Comparison of deceased patients with DNR orders to those without orders revealed no significant differences in the proportion of patients that received antibiotic therapy (87.9% vs. 84.2%, P = 0.599) or the number of days of antibiotic use (median 13 [IQR 6–22] vs. 14 [IQR 4–23] days). Malignancy was the most common cause of death (62.4%), and 21 patients (6.9%) received cardiopulmonary resuscitation during admission.

**Table 2:** Death-related characteristics of patients who died in the general medical wards.

Variable	Died patients (N = 303)
Infection-related death (%)	86 (28.4)
Presence of DNR order (%)	265 (87.5)
Time from first record of DNR order to death (days), median (IQR)	2 (1–6)
Antibiotic use after DNR (%)	158/265 (59.6)
Duration of antibiotic use after DNR, median (IQR)	2 (0–5)
CPR during admission (%)	21 (6.9)

CPR, cardiopulmonary resuscitation; DNR, do not resuscitate; IQR, interquartile range.

As shown in Table 3, bacterial infections were more common in patients who died than in those who survived (57.8% vs. 40.6%, P<0.001). Antibiotic therapy lasting more than 24 hours was also more common in deceased than in surviving patients (87.5% vs. 65.7%, P<0.001). The median number of antibiotic therapy courses were two (IQR 1–3) and one (IQR 0–2) for deceased and surviving patients, respectively. Third-generation cephalosporins were the most commonly prescribed antibiotics in both patient groups (43.9% and 39.3%). However, antibiotics were used for significantly longer periods in deceased patients than in those who survived (median 13 [IQR 5–23] vs. 7 [IQR 0–18] days, P<0.001). MDROs were more commonly isolated in deceased patients than in surviving patients (25.7% vs. 10.6%, P<0.001).

**Table 3:** Comparison of antibiotic use and MDRO isolation data of deceased and surviving patients.

Variable	Died patients (N = 303)	Survived patients (N = 303)	P value
**Bacterial infection during admission (%)**	175 (57.8)	123 (40.6)	<0.001
**Bacterial infection at time of death or discharge (%)**	157 (51.8)	32 (10.6)	<0.001
lung	81 (26.7)	8 (2.6)	
abdomen	37 (12.2)	15 (5.0)	
urinary tract	12 (4.0)	3 (1.0)	
skin and soft tissue	8 (2.6)	3 (1.0)	
central venous catheter	3 (1.0)	0	
bone and joint	0	1 (0.3)	
undefined fever	16 (5.3)	2 (0.7)	
**Antibiotic therapy for >24 h during admission (%)**	265 (87.5)	199 (65.7)	<0.001
**Number of antibiotic therapy courses administered per patient, median (IQR)**	2 (1–3)	1 (0–2)	<0.001
**Antibiotic class used (%)**			
β-lactam/β-lactamase inhibitors	121 (39.9)	56 (18.5)	<0.001
first-generation cephalosporins	10 (3.3)	22 (7.3)	0.029
second-generation cephalosporins	4 (1.3)	3 (1.0)	0.724
third-generation cephalosporins	133 (43.9)	119 (39.3)	0.249
fourth-generation cephalosporins	30 (9.9)	13 (4.3)	0.007
carbapenems	81 (26.7)	33 (10.9)	<0.001
aminoglycosides	12 (4.0)	15 (5.0)	0.555
glycopeptides	46 (15.2)	26 (8.6)	0.012
fluoroquinolones	71 (23.4)	49 (16.2)	0.025
macrolides	17 (5.6)	22 (7.3)	0.408
metronidazole	66 (21.8)	44 (14.5)	0.020
tigecycline	1 (0.3)	3 (1.0)	0.624
colistin	6 (2.0)	2 (0.7)	0.177
**Antibiotic use days, median (IQR)**	13 (5–23)	7 (0–18)	<0.001
**Number of MDROs isolated per patient (%)**			
1	60 (19.8)	27 (8.9)	
2	10 (3.3)	4 (1.3)	
3	2 (0.7)	0	
4	1 (0.3)	0	
**Isolation of MDROs during admission (%)**	78 (25.7)	32 (10.6)	<0.001
MRSA	32 (10.6)	10 (3.3)	<0.001
VRE	12 (4.0)	3 (1.0)	0.033
TGC-resistant Enterobacteriaceae	33 (10.9)	14 (4.6)	0.004
carbapenem-resistant non-fermenters	21 (6.9)	9 (3.0)	0.025

IQR, interquartile range; MDRO, multidrug-resistant organism; MRSA, methicillin-resistant *Staphylococcus aureus*; TGC, third-generation cephalosporin; VRE, vancomycin-resistant enterococci.

Comparison of deceased patients who had MDROs during their admissions to those who did not (Table 4) revealed that bacterial infections were more common in deceased patients with MDROs than in those without MDROs (89.7% vs. 46.7%, P<0.001). The proportion of patients receiving antibiotic therapy was higher in deceased patients with MDROs than in those without MDROs (98.7% vs. 83.6%, P<0.001). The number of antibiotic therapy courses administered was also higher (median 3 vs. 2 courses, P<0.001), and antibiotics were used for longer periods of time in deceased patients with MDROs than in those without MDROs (median 22 vs. 10 days, P<0.001). DNR orders were less common in deceased patients with MDROs (79.5% vs. 90.2%, P = 0.014). In multivariable analyses, factors including bacterial infection during admission (AOR 6.19, 95% CI 2.67–14.39), biliary drainage tube (AOR 3.50, 95% CI 1.02–12.01), nasogastric or gastrostomy tube (AOR 3.08, 95% CI 1.56–6.09), and longer duration of antibiotic use (AOR 1.04, 95% CI 1.02–1.06) were associated with deceased patients who had MDROs during their admissions.

**Table 4:** Comparison of characteristics of patient with and without MDROs among 303 deceased patients.

Variable	Patients with MDROs (N = 78)	Patients without MDROs (N = 225)	P value	Unadjusted odds ratio (95% CI)	Adjusted odds ratio (95% CI)
**Male**	47 (60.3)	140 (62.2)	0.758	0.92 (0.54–1.56)	
**Age ≥65 years**^a^	55 (70.5)	128 (56.9)	0.034	1.81 (1.04–3.15)	1.92 (0.94–3.93)
**Bacterial infection during admission (%)**^a^	70 (89.7)	105 (46.7)	<0.001	10.00 (4.60–21.75)	5.56 (2.10–14.74)
**Underlying disease (%)**					
solid tumor^a^	46 (59.0)	183 (81.3)	<0.001	0.33 (0.19–0.38)	0.29 (0.14–0.61)
cardiovascular disease	36 (46.2)	94 (41.8)	0.501	1.20 (0.71–2.00)	
diabetes mellitus	27 (34.6)	56 (24.9)	0.097	1.60 (0.92–2.79)	
neurologic disease	18 (23.1)	32 (14.2)	0.069	1.81 (0.95–3.45)	
chronic liver disease	9 (11.5)	40 (17.8)	0.197	0.60 (0.28–1.31)	
chronic obstructive pulmonary disease	12 (15.4)	23 (10.2)	0.219	1.60 (0.75–3.39)	
hematologic disease	10 (12.8)	15 (6.7)	0.089	2.06 (0.88–4.80)	
biliary/pancreatic disease	5 (6.4)	14 (6.2)	0.953	1.03 (0.36–2.97)	
**Predisposing conditions (%)**					
indwelling urethral catheter^a^	50 (64.1)	86 (38.2)	<0.001	2.89 (1.69–4.93)	0.93 (0.43–1.99)
central venous catheter^a^	29 (37.2)	45 (20.0)	0.002	2.37 (1.35–4.16)	1.85 (0.87–3.94)
anticancer chemotherapy	13 (16.7)	56 (24.9)	0.136	0.60 (0.31–1.78)	
nasogastric or gastrostomy tube^a^	31 (39.7)	30 (13.3)	<0.001	4.29 (2.37–7.77)	2.59 (1.24–5.41)
immunosuppressive therapy	5 (6.4)	17 (7.6)	0.737	0.84 (0.30–2.35)	
Neutropenia	9 (11.5)	16 (7.1)	0.221	1.70 (0.72–4.03)	
biliary drainage tube^a^	8 (10.3)	6 (2.7)	0.006	4.17 (1.40–12.43)	4.25 (1.23–14.68)
**Presence of DNR order**^a^	62 (79.5)	203 (90.2)	0.014	0.42 (0.21–0.85)	0.47 (0.19–1.12)
**Antibiotic therapy for >24 h during admission (%)**^a^	77 (98.7)	188 (83.6)	<0.001	15.15 (2.04–112.41)	2.37 (0.26–22.06)
**Number of antibiotic therapy courses administered per patient, median (IQR)**^a^	3 (2–4)	2 (1–3)	<0.001	1.63 (1.35–1.96)	0.92 (0.66–1.29)
**Days of antibiotic use, median (IQR)** ^a^	22 (14–34)	10 (3–17)	<0.001	1.05 (1.03–1.07)	1.05 (1.02–1.08)

CI, confidence interval; DNR, do not resuscitate; IQR, interquartile range; MDRO, multidrug-resistant organism.

^a^ Included in multivariable logistic regression analysis.

## Discussion

This study revealed extensive antibiotic exposure before death among deceased patients, even those who had provided DNR orders, in the general medical wards of acute care hospitals. Isolation of MDROs was more common in patients who died than in those who survived. Longer antibiotic use duration was associated with dying patients who had MDROs during admission. These findings support our hypothesis but a causality between the antibiotic exposure and isolation of MDROs in the deceased patients could not be defined because of limitations of this study (refer to below).

After opioid analgesics, antibiotics are the most commonly administered drugs on the day of death in terminally ill patients, and many of these patients receive antibiotics until death in Korea [5]. In this study, about 90% of patients who died in the general internal medicine wards received at least one course of antibiotic therapy during their hospital stay. Given the proportion of patients with bacterial infection who received antibiotic therapy during admission, antibiotics might be abused at some point during admission in about 30% of deceased patients. It has not been determined whether the use of antibiotics for end-of-life treatment is appropriate and beneficial in terms of improving symptoms in terminally ill patients [7,12]. Furthermore, aggressive treatment of infection has not been found to alter the underlying disease process or improve survival rates among patients with terminal illness [16]. Considering the risk of antibiotic resistance and the paucity of novel antibiotics in the development pipeline, the use of antibiotics in terminally ill patients should be rationalized [16].

Indiscriminate use of antibiotics in terminally ill patients may cause unintended consequences, including individual patient burdens as well as burdens extending to other patients in the same healthcare setting through the possible selection for antibiotic resistance [4]. Although the deceased patients with MDROs had more bacterial infections during admission in this study, multivariable analyses indicated that longer duration of antibiotic use was associated with the isolation of MDROs in these patients. This result is consistent with previous findings that antibiotic courses of longer duration are associated with higher rates of resistance [17]. MDROs that colonize or infect deceased patients can contaminate the environment or the hands of healthcare workers and spread to surviving patients or vice versa [9,11]. Therefore, careful selection of indications for and duration of antibiotic use at the end of life is important to prevent the development and dissemination of antibiotic resistance.

In Asia, physicians are typically more aggressive in their end-of-life treatment compared with their Western European counterparts due to cultural differences [18]. They are less likely to withhold or withdraw life-sustaining treatment, and they will initiate antibiotic treatment in terminally ill patients [18]. In this study, DNR consent was obtained from 87.5% of 303 patients who died in general medical wards in Korea. Among those deceased patients with DNR orders, 59.6% received antibiotic therapy even after providing a DNR order, although some of them had bacterial infections at the time of death. In addition, antibiotics were administered to patients who died regardless of the presence of DNR orders. This is consistent with the view that physicians may feel more comfortable in continuing to try to correct a theoretically reversible condition using antibiotics even in the face of an irreversible dying process [19].

This study has some limitations. First, we investigated the isolation of MDROs based on the results of clinical and hospital surveillance cultures limited to the hospital stay during the study period. Since the microbiology data before this admission and data on the time of isolation for each MDRO were not collected, we could not discriminate whether the MDROs isolated during this admission were already colonized before this admission or newly acquired. As a result, we could not determine the causal relation between the antibiotic use and acquisition of MDROs in the study patients. Second, since we included all patients who died in the internal medical wards, and not just those who were terminally ill, there may be differences in the population of deceased patients compared to previous studies. However, because almost 90% of patients had DNR orders, this difference was likely slight. Third, no was made distinction between colonization and infection. We did not determine the appropriateness of the antibiotic therapy administered to the study patients, so we were unable to estimate the proportion of controlled antibiotic use in patients who died in the general medical wards. Nevertheless, based on the finding in this study that many of the deceased patients had MDROs during their hospital stay before their deaths, it is likely that these patients with MDROs could be sources of transmission to other patients.

In conclusion, our study found that a substantial proportion of patients who died in the general medical wards of acute care hospitals were exposed to antibiotics before death. Antibiotic prescription was common even after obtaining their DNR orders in those deceased patients. In addition, the isolation of MDROs during their hospital stay was more common in patients who died than in those who survived. Because these patients may represent reservoirs of resistant bacteria that could be transmitted to surviving patients in hospital settings [8,9], strategies for judicious antibiotic use and appropriate infection control should be applied to this patient population. Further prospective studies are needed to evaluate whether overuse or abuse of antibiotics in the dying patients in general medical wards contributes to the acquisition and transmission of MDROs during their hospital stay.
